# Efficacy of Topical Treatments for the Management of Symptomatic Oral Lichen Planus: A Systematic Review

**DOI:** 10.3390/ijerph20021202

**Published:** 2023-01-10

**Authors:** Giorgio Serafini, Alberto De Biase, Luca Lamazza, Giulia Mazzucchi, Marco Lollobrigida

**Affiliations:** Department of Oral and Maxillo Facial Sciences, “Sapienza” University of Rome, Via Caserta 6, 00161 Rome, Italy

**Keywords:** oral lichen planus, erosive-atrophic lichen planus, OLP, topical drugs, topical medications, corticosteroids, topical calcineurin inhibitors, systematic review

## Abstract

Oral lichen planus (OLP) is a chronic mucosal inflammatory disease associated with T-cell-mediated immunological dysfunction. Symptomatic OLP is a painful condition, and complete healing is often not achieved. The aim of this systematic review was to assess the effectiveness of topical drugs, medications, and other interventions compared to placebo or to other treatments in pain reduction and clinical resolution in adult patients with symptomatic OLP. A detailed electronic literature search was performed through the MEDLINE (PubMed) database between 1 January 2005 and 30 September 2022. Eligible studies were selected based on the inclusion criteria, and a quality assessment was conducted. From 649 titles, 121 articles were selected as abstracts, 75 papers were assessed as full text, along with 15 other papers obtained through a manual search. A total of 15 RCTs were finally included in the review process. Because of the significant heterogeneity in the study design of the included studies, no meta-analysis of the data could be performed. Topical corticosteroids represent the first-line treatment in the management of symptomatic OLP due to their efficacy and minimal adverse effects. Calcineurin inhibitors seem to be equally effective and are indicated in recalcitrant cases, extensive lesions, patients susceptible to oral candidiasis, or cases unresponsive to corticosteroids. Other treatments, such as aloe vera, chamomile, isotretinoin, ozone, and laser therapy, could be beneficial as adjunct therapies in association with first-line treatments.

## 1. Introduction

Oral lichen planus (OLP) is a chronic inflammatory disorder associated with T-cell-mediated immunological dysfunction with an unclear etiology [1]. In fewer cases, etiological factors can be identified, including drugs, dental materials (e.g., amalgam), and infectious agents (e.g., hepatitis C virus infection) [2,3].

Lichen Planus (LP) involves approximately 1% to 2% of the population, affecting the skin and/or any lining mucosa [4]. Although LP may occur in all age groups, women aged between 30 and 60 years are more likely to have this condition [5].

LP is often found in the oral cavity with different lesion patterns, including reticular, papular, plaque-like, atrophic, and ulcerative (erosive) lesions [6,7]. The most involved sites are the buccal mucosa, borders, and dorsum of the tongue and gingiva. The bilateral and symmetric distribution is typical of OLP. The hard and soft palate, lips, and floor of the mouth are rarely affected. The reticular pattern is the most frequent and consists of a network of overlapping white threads, referred to as Wickham’s striae, which are rarely symptomatic [8]. The ulcerative (erosive) and atrophic patterns can affect any mucosal surface, including the buccal mucosa, tongue, and gums, causing varying degrees of symptoms ranging from a burning sensation to severe pain and difficulty in eating, significantly impairing the quality of life [9,10].

Clinical manifestations of OLP are often sufficient to make a correct diagnosis. However, for erosive and atrophic OLP, an oral biopsy with histopathological analysis may be recommended to confirm the clinical diagnosis and exclude dysplasia or malignancy [11,12,13]. Moreover, several authors have observed that patients with longstanding atrophic and ulcerative OLP have an increased risk (up to 5.6%) of developing oral squamous cell carcinoma (OSCC) [14,15,16,17].

Erosive and atrophic OLP often require active treatment to achieve pain relief and to promote mucosal healing. The usual treatment is based on topical corticosteroids (TCSs) (e.g., clobetasol propionate (CP), betamethasone, dexamethasone, triamcinolone acetonide (TA), prednisolone, fluocinolone) applied to the painful areas. As a general guideline, topical corticosteroids are indicated for mild to moderately symptomatic OLP, while systemic treatments are reserved for more severe cases. Other treatments have also been proposed [16], such as topical calcineurin inhibitors (TCIs), e.g., pimecrolimus, tacrolimus (TAC), ciclosporin; nutraceuticals, e.g., aloe vera (AV), lycopene, purslane, ignatia, curcumin, quercetin); retinoids, e.g., tretinoin, isotretinoin, tazarotene; systemic immunosuppressant, e.g., mycophenolate mofetil, methotrexate, azathioprine, dapsone; immunostimulants, e.g., thalidomide, levamisole; biological agents like TNF-a inhibitors, e.g., infliximab, adalimumab, etanercept, rituximab, alefacept, efalizumab, basiliximab, and BCG-PSN; novel therapies including hyaluronic acid, amitriptyline, and amlexanox [18]. In addition to these pharmacological treatments, ozone therapy, cryotherapy with nitrous oxide gas (NOG), photodynamic therapy (PDT), and low-level laser therapy (LLLT), also called photobiomodulation (PBM), have been proposed for patients with symptomatic OLP. With respect to the effectiveness of all the above-mentioned treatments, no conclusive results have been achieved so far [19,20,21].

This study aimed to systematically assess the evidence from randomized controlled clinical trials (RCTs) on the effectiveness of topical drugs, medications, and other interventions in achieving pain relief and clinical resolution of symptomatic OLP in adults.

## 2. Methods

This manuscript was prepared according to the guidelines of the Preferred Reporting Items for Systematic Reviews and Meta-Analyses (PRISMA) statement [22], and the review protocol was registered on PROSPERO (registration number: CRD42021235061).

The focused PICO question was “what is the efficacy of topical drugs, medications, and interventions on pain relief and clinical resolution in adult patients with symptomatic OLP?”.

Pain relief and clinical resolution of symptomatic OLP were considered primary outcomes. The secondary outcome was the incidence of adverse effects associated with the treatments.

### 2.1. Information Sources and Search Strategy

A systematic review was conducted by searching an electronic database (MEDLINE, PubMed) to locate all relevant articles published in peer-reviewed journals between 1 January 2005 and 30 September 2022. To perform the research, the following key terms were applied as text words to the database search string: (“oral” OR “mouth”) AND (“lichen planus” OR “LP”) AND (“treatment” OR “management” OR “therapy” OR “therapeutic”) AND (“topic” OR “topical” OR “steroid” OR “corticosteroid”). The reference lists of the articles in previous systematic reviews and meta-analyses were also manually searched for additional relevant papers. To find additional literature, a manual search was conducted through the following international journals up to 30 September 2022: Journal of Oral Pathology and Medicine, Oral Diseases, and Oral Surgery Oral Medicine Oral Pathology Oral Radiology.

### 2.2. Eligibility Criteria

The following eligibility criteria were used:

Inclusion criteria:

(1) Randomized controlled clinical trials (RCTs) or controlled clinical trials
(2) Publications in English
(3) Clinical studies on adults only
(4) Clinical and/or histological diagnosis of OLP
(5) Population: adult patients with symptomatic OLP
(6) Intervention: active topical drugs, topical medications, and other non-drug topical therapies
(7) Comparison: placebo or another active topical drug or medication
(8) Reporting of pain improvement (VAS), clinical resolution (as assessed by different clinical scores), and adverse effects
(9) At least 30 participants per group in the randomization

Exclusion criteria:

(1) Review articles, meta-analysis, cohort studies, retrospective studies, observational studies, case series, case reports, professional opinions
(2) Publications not in English
(3) Animal and in vitro studies
(4) Studies not reporting the outcomes of interests
(5) Associated systemic therapies
(6) Refractory or unresponsive OLP cases
(7) Oral lichenoid lesions

### 2.3. Screening and Selection of Studies

Screening of the titles and selection of abstracts for potential inclusion in the review was carried out independently by two reviewers (GS and ML). Studies were selected for full-text reading if: (i) the title, the abstract, or both included search keywords and the information related to the eligibility criteria; (ii) relevant titles but the abstracts did not include information concerning the eligibility criteria; (iii) relevant titles but without abstracts. The full-text papers were read thoroughly to choose those that fulfilled the eligibility criteria. Any disagreement was resolved by discussion between the two reviewers. The Kappa value for inter-reviewer agreement during the screening of the title and abstract was evaluated.

### 2.4. Data Extraction and Analysis

Data of interest from all included studies were extracted, using a specially designed form, by the same two reviewers (GS and ML) and analyzed by the other two authors (GM and LL).

Primary outcomes (pain improvement, clinical resolution), secondary outcomes (adverse effects), interventions, and other data of interest were extracted from the selected articles and reported in the form. Regarding pain improvement, visual analogue scale (VAS) values at baseline and at the end of treatment were reported, highlighting the significance of any reduction in the statistical analysis reported. The clinical resolution means the improvement of patients’ clinical signs (reduction in lesion size, reduction in erosive area, reduction of erythematous and ulcerative lesions as assessed by different scores reported in the literature) reporting the number of patients who had a complete resolution (if possible) and the significance of any reduction in the statistical analysis reported. Adverse effects are unexpected or unwanted effects linked to the action of the topical therapy used in the included studies.

### 2.5. Quality Assessment

The Cochrane Collaboration’s risk of bias assessment tool has been used to assess the quality of the included Studies [23].

Assessment of the studies’ methodological quality was performed by two reviewers (GS and ML), who reported their judgments supported with an explanation by mentioning the relevant quotes from the studies (if possible).

For each included study, the following domains were assessed:

- Random sequence generation
- Allocation concealment
- Blinding of participants and personnel
- Blinding of outcome assessment
- Incomplete outcome data
- Selective reporting
- Group imbalance
- Sample size
- Follow-up period
- Conflict of interest

For each domain, a judgment of “low”, “high”, or “unclear” risk of bias was assigned. Studies were classified into the following categories: “low risk of bias” if low risk for all key domains was reported; “unclear risk” of bias if unclear risk for one or more key domains was reported; “high risk” of bias if high risk of one or more key domains was reported. Any disagreement was resolved by discussion between the two reviewers.

### 2.6. Statistical Analysis

Because of the different study designs, dosages, and timing of administration of topical treatments in the selected studies, no meta-analysis of the data could be performed.

## 3. Results

**Figure 1:**
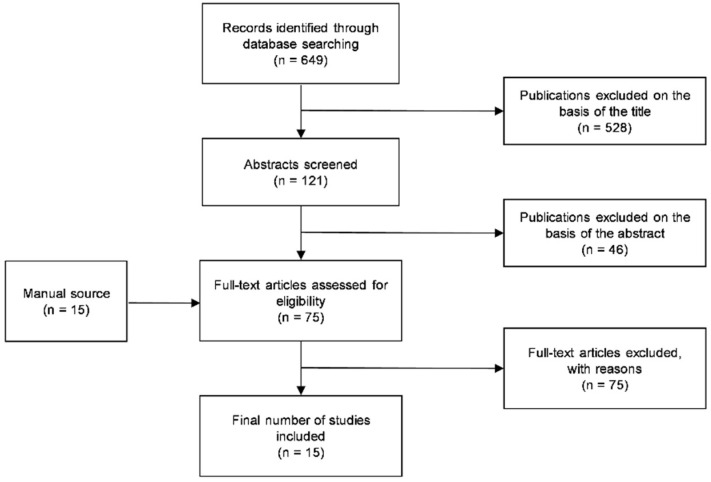
The PRISMA flowchart of the study selection process.

The Medline search yielded 649 references. After the titles’ review, 121 abstracts were screened, and among these, 75 potentially pertinent articles were selected for full-text reading, in addition to 15 papers obtained through manual search. The Kappa value for inter-reviewer agreement during the screening of the title and abstract was 0.88. Of these studies, 15 fulfilled the inclusion criteria and were evaluated. The excluded studies are recorded in Table S1, reporting the reasons for exclusion. The PRISMA flowchart outlining the papers’ selection process is shown in Figure 1.

### 3.1. Characteristics of Included Studies

The characteristics of the included studies are reported in Appendix A. All included studies were RCTs [24,25,26,27,28,29,30,31,32,33,34,35,36,37,38]. Ten trials had a two-arm parallel design [25,26,28,30,31,32,33,34,35,37], two trials had a four-arm parallel design [27,29], and three had a two-arm split-mouth design [24,36,38]. The total number of randomized participants in the trials was 1074, of whom 1025 completed the follow-up, with a mean of approximately 68 participants per study, with the number per study ranging from 23 to 137.

Three trials compared an active intervention with a placebo [31,33,36], while ten trials compared two active treatments [24,25,26,28,30,32,34,35,37,38]. In a four-arm trial, three different concentrations of the same active treatment were compared with each other and with a placebo [27]. In the other four-arm trial, three active treatments were compared with each other and with a placebo [29].

TCSs were used in eleven trials, including the following molecules: TA [24,25,30,32,36,37,38], CP [27,28], dexamethasone [29,35], and betamethasone [30]. TCIs were administered in four trials, including TAC [25,28,32] and ciclosporin [37]. Other topical treatments included cryotherapy with NOG [24], LLLT [26,29], PDT [38], ozone therapy [29], chamomile [31], AV [26,33], isotretinoin [34], and thalidomide [35].

In a split-mouth RCT [24], Amanat et al. compared the effects of cryotherapy with NOG on one side of the lesion with topical 0.1% TA ointment in Orabase^®^ (Convatec, Reading, UK) on the other side. A significant pain reduction was observed in both groups. The difference between the groups was significant after 2 and 4 weeks but not after 6 weeks. Regarding clinical resolution, the results were similar: a complete resolution occurred in 8.70% (2 of 23) of the TA-treated patients and in 21.74% (5 of 23) of the cryotherapy-treated patients. Seventeen patients reported minor swelling at the sites treated with cryotherapy, and twelve patients experienced increased pain in the first 7–10 days after treatment.

Azizi et al. [25] compared TA ointment (10 mg/mL TA in Orabase^®^) with 0.1% TAC ointment. After four weeks of treatment, both groups showed an improvement in terms of pain reduction and severity score of lesions compared to the baseline, but only pain reduction was statistically significant. No significant differences were observed in the intergroup analysis for both pain reduction and clinical scores.

In the study by Bhatt et al. [26], patients were treated for two months with either AV gel (500 mg powder mixed with carboxymethylcellulose powder and distilled water) or LLLT (980 nm diode laser). At the end of treatment and during the follow-up, both groups showed a significant improvement compared to the baseline in pain relief and clinical scores. The patients treated with LLLT also showed significantly better results compared to the AV group in terms of VAS, site score, and severity score at the end of treatment, while no intergroup differences were noted during the follow-up period.

In a multicenter study, Brennan et al. [27] assessed three different doses (1, 5, 20 μg) of a CP mucoadhesive patch in comparison to a placebo (no treatment). The 20-μg group achieved the most relevant and statistically significant improvement compared to the placebo with respect to the ulcer area, symptom severity, disease activity, pain, and quality of life. The most reported adverse effects included candidiasis, application site pain or infection, and salivary hypersecretion.

In the study by Hettiarachchi et al. [28], patients were treated either with 0.1% TAC cream or 0.05% CP cream for 3 weeks. Significant changes in pain and clinical score were found in both groups after treatment. As for clinical resolution, the TAC group was significantly more effective than the clobetasol group. No adverse effects were observed in both groups.

Kazancioglu et al. [29] compared the influence of ozone, 808 nm-LLLT with diode laser, dexamethasone mouthwash, and placebo. Significant symptom relief was obtained after treatment for all groups of intervention, but with no intergroup differences. Clinical improvement was significant in the ozone (10%, 3 of 30 patients) and corticosteroid (20%, 6 of 30 patients) groups, but no significant changes were observed between the groups. No complications nor adverse effects were observed.

Liu et al. [30] assessed the efficacy of the intralesional compound betamethasone (5 mg betamethasone dipropionate and 2 mg betamethasone disodium phosphate per milliliter) compared to 40 mg/mL TA. The betamethasone group showed a significantly higher reduction in the erosive area compared to the TA group, while pain reduction was similar.

Lopez Jornet et al. [31] assessed the clinical efficacy of the topical administration of 2% chamomile gel versus a placebo. After 4 weeks of treatment, patients treated with chamomile showed significant pain improvement compared with the baseline assessment, while no improvement was observed in the placebo group. A complete resolution of the symptoms was achieved in 19.23% (5 of 26) of patients in the chamomile group, while no patient (0 of 29) in the placebo group had a complete response. Significant changes in clinical scores were also observed in the experimental group (*p* < 0.001) after treatment. No adverse effects were observed in either of the groups during the study.

Manjunatha et al. [32] compared the efficacy of topical 0.1% TAC with 0.1% TA. Both molecules were administered through a protective paste (Orabase^®^). The authors observed a significantly higher improvement of symptoms in the TAC group compared to the TA group. As for clinical resolution, a significantly better response was observed for the TAC group compared to the TA group B (*p* = 0.002). In the TAC group, 63.33% (19 of 30) patients showed a complete resolution, against 6.67% (2 of 30) of the TA group. No significant adverse effects were noted during treatment and follow-up.

Salazar-Sánchez et al. [33] evaluated the efficacy of the topical application of AV gel compared with a placebo. No statistically significant differences were recorded between the groups with respect to pain sensation after treatment. In the AV group, complete pain remission was achieved in 61.29% (19 of 31) patients after treatment. In the placebo group, complete pain remission was achieved in 32.26% (10 of 31) patients after treatment. As for clinical resolution, no significant differences between the groups were observed after 6 and 12 weeks. No significant adverse effects were reported.

Scardina et al. [34] investigated the potential use of vitamin A derivatives by comparing the efficacy of two different doses (0.05% and 0.18%) of isotretinoin on erosive OLP. In this study, 0.18% isotretinoin resulted in a significantly more effective reduction of the lesions’ clinical score of atrophic-erosive OLP. The authors then encouraged the use of the higher concentration instead of the more commonly used 0.05% concentration.

Wu et al. [35] evaluated the short-term efficacy and safety of topical 1% thalidomide paste compared with 0.043% dexamethasone paste. After a 1-week application, both groups showed significant reductions in VAS scores. A significant improvement in erosive size was also observed in both groups. Complete healing occurred in 54.55% (18 of 33) of thalidomide-treated patients and 56.67% (17 of 30) dexamethasone-treated patients. At intergroup analysis, however, no significant differences in erosion size and VAS were observed. None of the patients had severe systemic or topical adverse reactions.

In a split-mouth study, Xia et al. [36] compared the efficacy of a single intralesional injection of 0.5 mL TA (40 mg/mL) with no treatment (control group) by assessing pain relief (VAS) and lesional area reduction. The experimental group showed a significantly higher improvement compared to the control group with respect to ulcerative and erythematous areas and VAS at different time points. Both symptoms and signs were significantly reduced in the experimental group (*p* < 0.05) when comparing the 1-week follow-up to the baseline and the 2-week follow-up to the 1-week follow-up. No complications were observed after the intralesional injections.

Yoke et al. [37] have compared the efficacy of 100 mg/mL cyclosporine solution with 0.1% TA. No statistically significant differences were found between the groups, though a worsening in the clinical outcome was observed in the cyclosporine group.

In their split-mouth study, Zborowski et al. [38] assessed the efficacy of PDT compared to 0.05% TA. In this trial, 5% methylene blue (MB) and 0.05% TA were administered through dry porous polymer carriers. Following treatment, 33.3% of the patients in the PDT group and 22.2% of the patients in the TA group showed complete remission. After 3 months, 54.2% of patients in the PDT group and 62.9% of patients in the TA group had complete remission. Pain, lesions’ size, and clinical score significantly decreased in both groups, but no significant differences were found between the groups.

### 3.2. Quality Assessment of the Included Studies

Table 1 summarizes the risk of bias assessment of the included studies. Only one [28] of the fifteen included RCTs met all the risk of bias criteria and can therefore be assessed as at “low risk” of bias overall. Four studies [26,34,35,37] had one or more domains assessed as unclear and are deemed at “unclear risk” of bias overall. The remaining ten studies [24,25,27,29,30,31,32,33,36,38] had one or more domains at significant risk of bias (no blinding, selective reporting, or another risk of bias) and are therefore described as at “high risk” of bias overall.

Regarding the randomization, in nine trials [27,28,30,31,34,35,36,37,38], the method of randomization was considered adequate in both its components (sequence generation and allocation concealment); in three trials [26,29,33], sequence generation was adequate, but allocation concealment was unclear, and in the remaining three trials [24,25,32], sequence generation and allocation concealment were both unclear.

Regarding blinding, performance bias (blinding of participants and personnel) and detection bias (blinding of outcome assessment) were considered. Six trials [27,28,31,33,35,37] were judged at low risk of performance bias as both participants and personnel were blinded; three trials [25,26,34] were judged at unclear risk of performance bias, and six trials [24,29,30,32,36,38] were judged at high risk of performance bias. Five studies [27,28,29,30,38] reported that outcome assessment was blind and then considered at low risk of detection bias; two trials [24,32] were at high risk of detection bias, and eight studies [25,26,31,32,33,34,35,36,37] were at unclear risk.

As regards incomplete data outcome, thirteen trials [25,26,27,28,29,30,31,32,34,35,36,37,38] were judged at low risk of attrition bias since all enrolled participants completed the study, or the number of participants lost was not likely to have a clinically relevant impact on the intervention effect estimate. Two trials [24,33] were judged at high risk of attrition bias since the rate of dropouts per group was higher than 20%.

Regarding selective reporting, all fifteen trials [24,25,26,27,28,29,30,31,32,33,34,35,36,37,38] were judged at low risk of bias since all planned outcomes were reported.

Other sources of bias included group imbalance, sample size, follow-up period, and conflicts of interest. Regarding group imbalance, all fifteen trials [24,25,26,27,28,29,30,31,32,33,34,35,36,37,38] were judged at low risk of bias. As for sample size, all fifteen trials [24,25,26,27,28,29,30,31,32,33,34,35,36,37,38] were judged at low risk of bias (>30 participants per group in the randomization). Considering the follow-up period, eight trials [26,28,29,30,34,35,37,38] were at low risk of bias (follow-up > two months), while seven trials [24,25,27,31,32,33,36] were judged at high risk (follow-up < two months). Finally, regarding possible conflict of interest, ten trials [24,26,28,35,36,37,38] were judged at low risk of bias (authors declare no conflict of interest), one trial [27] was judged at high risk of bias (authors received funding from the pharmaceutical company who actively participated in the study), and four trials [25,29,33,34] were at unclear risk due to insufficient information.

### 3.3. Excluded Studies

Of the 90 full-text articles assessed, 75 were excluded from the final analysis due to the following reasons:

- Inadequate sample size
- Insufficient data to assess outcomes
- No RCTs
- Associated systemic therapy
- Included patients without symptoms
- Papers of methodology (protocol study)
- Only systemic therapy for OLP

The excluded studies were recorded in Table S1.

## 4. Discussion

The purpose of this systematic review was to assess the effectiveness of topical treatments in reducing pain and achieving clinical resolution in adult patients with symptomatic OLP. Therapies included both pharmacological (corticosteroids, calcineurin inhibitors, chamomile, AV, isotretinoin, and thalidomide) and non-pharmacological interventions (cryotherapy, ozone therapy, PDT, and LLLT).

TCSs are the most common treatment in the included studies. Corticosteroids are prescribed for a wide range of conditions due to their anti-inflammatory properties, including the reduction in the number and function of various immune cells, such as T and B lymphocytes, monocytes, neutrophils, and eosinophils. The most common adverse effect of the prolonged use of corticosteroids is an increased risk of acute pseudomembranous candidiasis [39]. Over the years, several TCSs have been used with different dosages and timing of administration. Commonly used TCSs include 0.025–0.05% CP gel 2–4 times daily, 0.1% TA ointment three times daily, 0.01% fluocinolone acetonide ointment 2–6 times daily, 0.1–0.15% dexamethasone solution or 0.043% dexamethasone paste three times daily. Less commonly used TCSs include fluticasone propionate spray 4 times daily and 0.1% betamethasone sodium phosphate solution, cream, or valerate aerosol 4 times daily. In this systematic review, TCSs were used in 11 of 15 studies: TA in seven trials [24,25,30,32,36,37,38], CP in two trials [27,28], dexamethasone in two trials [29,35], and betamethasone in one trial [30]. In 4 of these 11 studies, TCSs were used as a control group, and in 7 trials, TCSs were used as a test group. In these studies, TCSs were demonstrated to be effective in reducing the signs and symptoms of OLP. However, none of the included studies found major differences between the different molecules. In this regard, Yuan and colleagues [40] reported dexamethasone, TA, and betamethasone as equally recommendable with respect to efficacy and safety.

TCIs are considered second-line drugs in the treatment of symptomatic OLP. Calcineurin inhibitors are immunosuppressant drugs that inhibit the action of calcineurin, an enzyme that plays a key role in cell-mediated immunity. Calcineurin inhibitors exercise their immunosuppressive effects by inhibiting the expression of interleukins [41]. Commonly used TCIs include 0.1% TAC ointment 3 times daily, 1% pimecrolimus cream twice daily, and 100 mg/mL cyclosporine solution 3 times daily. Today, TAC and pimecrolimus are preferred to cyclosporine thanks to their higher potency. The adverse effects of the prolonged use of calcineurin inhibitors include transient burning sensation, high rates of relapse, and an increased risk for OSCC after long-term therapy [42]. For these reasons, TCIs are not recommended as first-line therapy for OLP but in patients unresponsive to TCSs or susceptible to oral candidiasis. The absence of dysplasia must be verified on histology before the administration of TAC, and strict follow-up is mandatory during the therapy and after its completion. In this review, one study [32] reported superior effects of 0.1% TAC compared to 0.1% TA, while Azizi and colleagues [25] reported similar effects. Similarly, Hettiarachchi and colleagues [28] have observed better clinical responses of 0.1% TAC compared to 0.05% CP. Significantly, these authors propose to consider TAC as a first-line therapy. These results agree with the meta-analysis of Da Silva et al. [43], who found similar beneficial effects of TAC 0.1% and pimecrolimus 1% in comparison to TCSs.

Chamomile is a well-known medicinal plant with anti-inflammatory and antiseptic properties. Its use has been described for different inflammatory oral diseases, including gingivitis, mucositis, and aphthous stomatitis [44,45]. One study [31] compared the efficacy of the topical application of 2% chamomile gel to a placebo. The authors noted that patients treated with chamomile showed significant improvements after 4 weeks of treatment for pain, burning sensation, and clinical resolution compared with those treated with a placebo, with no adverse effects.

AV is a plant that contains many active components, including vitamins (A, C, E), enzymes, amino acids, salicylic acid, minerals, and sterols, along with other fatty acids [46]. AV has anti-inflammatory, antimicrobial (antibacterial, antifungal, antiviral), antioxidant, and analgesic properties; moreover, it accelerates wound healing by promoting the proliferation and migration of fibroblasts and keratinocytes [46,47]. In this systematic review, Salazar-Sánchez et al. [33] compared the efficacy of AV to placebo, reporting no significant differences in relation to pain relief. This is in contrast with the findings of Choonhakarn et al. [48], who reported significantly higher symptomatologic improvement of OLP in patients treated with AV compared to the placebo group, though without a follow-up period after treatment. Moreover, a systematic review and meta-analysis [49] reported comparable effects of AV and TA, with the advantage of the absence of adverse effects. However, the authors do recommend further studies with larger sample sizes and longer follow-ups. In conclusion, in accordance with a recent systematic review [50], the included studies seem to support the use of topical phytomedicines as a complementary treatment.

Thalidomide is currently used for treating several malignancies and HIV-related complications. Due to its anti-inflammatory and anti-immunological properties [51], thalidomide has also been proposed for the treatment of autoimmune and inflammatory disorders, including aphthous stomatitis, rheumatoid arthritis, and Crohn’s disease. In the study of Wu et al. [35], OLP patients were treated with either 1% thalidomide paste or 0.043% dexamethasone paste. The treatments showed the same efficacy in terms of both pain reduction and clinical resolution. Despite its beneficial effects, thalidomide is infamously known for its teratogenicity, which caused birth defects in thousands of children in the late 1950s and early 1960s. For this reason, the pharmacological properties of thalidomide and its analogues have long been unrecognized, and only recently, interest has developed for its use in several conditions [52]. Despite some interesting results, there is no conclusive data about thalidomide use for treating oral conditions, and in the authors’ opinion, it is advisable to use drugs with better-known safety profiles and indications.

Isotretinoin is a retinoid derivative characterized by important biological effects on the cell cycle, cell differentiation, survival, and apoptosis. In this review, Scardina and colleagues [35] suggest the topical use of 0.18% isotretinoin in all cases of atrophic-erosive OLP, in addition to those cases in which systemic corticosteroid treatment is contraindicated. It is important to consider the advantages of topical administration of isotretinoin, considering the risk of teratogenesis associated with Vitamin A derivatives. The authors also recommend a specific method of administration by applying the drug on a gauze and then applying the gauze to the lesion sites.

Cryotherapy is described in the literature for the treatment of symptomatic erosive OLP [53]. Cold therapy is used in medicine for the treatment of several lesions. Its effects on tissues depend upon the severity of freezing, varying from inflammation to tissue destruction. Compared to conventional surgery, cryosurgery has some advantages, including minimal or no bleeding, ease of application, favorable healing without scarring [54], destruction of only a selected volume of tissue, localized action with no systemic side effects, and low incidence of infection. The disadvantages are unpredictable swelling, lack of precision compared with scalpel surgery [54], and pain augmentation in the first 7–10 days post-operative. In this systematic review, one trial compared a single session of cryotherapy with NOG and 0.1% TA ointment [24]. The authors observed the same efficacy in terms of sign score, pain score, and severity of lesions for both groups, concluding that cryotherapy can be considered an alternative or adjuvant therapy in OLP patients, reducing the use of pharmacological treatments with systemic side effects [24].

LLLT has also been proposed as an alternative treatment for symptomatic OLP with minimal side effects [55]. LLLT has been reported to have biostimulatory, anti-inflammatory, analgesic, anti-infective, and anti-ablation effects [56,57]. LLLT can stimulate cell differentiation by enhancing wound healing and epithelization [58]. On the other hand, LLLT requires expensive equipment and specific training. The role of LLLT in the management of symptomatic OLP is controversial. In this review, one RCT [26] compared LLLT with AV. Particularly, LLLT-treated patients showed better VAS scores, site scores, and severity scores at the end of treatment compared to those of the AV-treated patients. In another RCT, Kazancioglu et al. [29] compared LLLT with TCS, reporting higher efficacy of dexamethasone mouthwash compared to LLLT. Other studies in the literature reported different results. For instance, Dillenburg et al. [59] found that LLLT with diode was more effective than 0.05% CP gel. Conversely, Ferri et al. [60] reported the same efficacy of LLLT and CP. A recent systematic review of the literature [61] concluded that there is weak evidence of the superiority of LLLT compared to TCSs. Considering the small number of studies and their discordant results, LLLT should not be considered as the first-line treatment for OLP but rather an adjuvant therapy.

PDT represents a relatively novel approach to several conditions. PDT combines three components: a photosensitizer (e.g., methylene blue), a light source with a specific wavelength (660 nm), and oxygen [62]. Briefly, methylene blue (MB) is accumulated in the target cells following topical administration. MB is activated by the laser light, releasing reactive oxygen species (ROS), which cause cellular damage, membrane lysis, and protein inactivation [63]. PDT has several advantages, including being minimally invasive, no scar formation, and no need for anesthesia, and it has shown promising results in the management of oral lesions. In this review, one split-mouth RCT [38] compared MB-PDT to topical application of 0.05% TA with no statistically significant differences observed between the groups. Other studies have highlighted the beneficial effects of PDT for treating OLP [64,65]. However, more research is needed to define the exact indications of PDT for OLP patients and clarify whether it should be considered as a first-line treatment or as an adjuvant therapy.

Ozone therapy has been proposed as a complementary approach in medicine and dentistry [66]. Ozone has a high oxidation potential and interacts with blood components, inducing immunomodulatory changes and stimulating blood microcirculation in tissues [67]. It also has antimicrobial properties, promotes wound healing, and contributes to pain relief. In this review, ozone was tested in only one RCT. In a four-arm trial, Kazancioglu et al. [29] compared three active treatments (LLLT with diode laser, ozone therapy using an ozone generator applied intraorally, dexamethasone mouthwash) with each other and with placebo, concluding that ozone and corticosteroid therapies are more effective than LLLT in the treatment of OLP.

The main limitation of this review is the lack of a meta-analysis due to the heterogeneity of the studies with respect to the different types, dosages, and times of administration of treatments. Moreover, the quality of the included studies was generally questionable for different aspects. The studies’ main limitations include the relatively small sample size, short duration of treatment, absent or inadequate follow-up after treatment, and the heterogeneous reporting of treatment outcomes. As for the sample size, even if RCTs with at least 30 patients per group were included in the review, this is far from representing a high statistical power. For this reason, the clinical relevance of the results should be considered with caution. Well-controlled double-blind RCTs with larger sample sizes, adequate duration, long-term follow-up, and uniform outcomes are needed to standardize and improve treatment options for atrophic-erosive OLP.

## 5. Conclusions

TCSs are effective as first-line treatment for mild to moderately symptomatic OLP due to their cost-benefit ratio. TCIs have similar efficacy to TCSs and are used for managing recalcitrant OLP, extensive lesions, and patients susceptible to oral candidiasis or unresponsive to corticosteroids. Long-term side effects of calcineurin inhibitors must be further investigated, including possible association with OSCC.

Based on available data, topical AV, chamomile, isotretinoin, ozone, and laser therapy should be considered adjuvant therapies in association with first-choice treatments.

## Figures and Tables

**Table 1:** Risk of bias assessment. (+, green colour) low risk, (-, red colour) high risk, (?, yellow colour) unclear risk.

	Random Sequence Generation	Allocation Concealment	Blinding of Participants and Personnel	Blinding of Outcome Assessment	Blinding of Outcome Assessment	Incomplete Data Outcome	Selective Reporting	Group Imbalance	Sample Size	Follow-Up Period	Conflict of Interest
Amanat et al. (2014) [24]	?	?	-	-	-	+	+	+	+	-	+
Azizi et al. (2007) [25]	?	?	?	?	+	+	+	+	+	-	?
Bhatt et al. (2022) [26]	+	?	?	?	+	+	+	+	+	+	+
Brennan et al. (2022) [27]	+	+	+	+	+	+	+	+	+	-	-
Hettiarachchi et al. (2017) [28]	+	+	+	+	+	+	+	+	+	+	+
Kazancioglu et al. (2015) [29]	+	?	-	+	+	+	+	+	+	+	?
Liu et al. (2013) [30]	+	+	-	+	+	+	+	+	+	+	+
Lopez Jornet et al. (2016) [31]	+	+	+	?	+	+	+	+	+	-	+
Manjunatha et al. (2012) [32]	?	?	-	-	+	+	+	+	+	-	+
Salazar-Sanchez et al. (2010) [33]	+	?	+	?	-	+	+	+	+	-	?
Scardina et al. (2006) [34]	+	+	?	?	+	+	+	+	+	+	?
Wu et al. (2010) [35]	+	+	+	?	+	+	+	+	+	+	+
Xia et al. (2006) [36]	+	+	-	?	+	+	+	+	+	-	+
Yoke et al. (2006) [37]	+	+	+	?	+	+	+	+	+	+	+
Zborowski et al. (2021) [38]	+	+	-	+	+	+	+	+	+	+	+

## Data Availability

The data presented in this study are available on request from the corresponding author.

## Supplementary Materials

The following supporting information can be downloaded at: https://www.mdpi.com/article/10.3390/ijerph20021202/s1, Table S1: Characteristics of excluded studies.

## List of Abbreviation

OLP	oral lichen planus
LP	lichen planus
OSCC	oral squamous cell carcinoma
TCSs	topical corticosteroids
CP	clobetasol propionate
TA	triamcinolone acetonide
TCIs	topical calcineurin inhibitors
TAC	tacrolimus
AV	aloe vera
NOG	nitrous oxide gas
PDT	photodynamic therapy
LLLT	low-level laser therapy
PBM	photobiomodulation
CHX	chlorhexidine
RCTs	randomized controlled clinical trials
PROSPERO	Preferred Reporting Items for Systematic Reviews and Meta-Analyses
VAS	visual analogue scale
MB	methylene blue

## Appendix A

**Table A1:** Characteristics of included studies (in alphabetical order).

Amanat 2014 [24]	
Study design	Split-mouth RCT
Country	Iran
Patients randomized (analyzed)	Group A: 30 (23) Group B: 30 (23)
Interventions	Group A: 0.1% TA ointment in Orabase^®^ (3 times daily for 1 week, twice daily on week 2, once daily on week 3, on alternate days on week 4 and discontinued at week 5) Group B: single session of 20–25 s of freeze-thaw cycles with NOG under local anesthesia
Pain improvement	Group A - VAS baseline (mean ± SD): 2.25 ± 2.35 - VAS end of treatment (mean ± SD): 1.28 ± 2.64 Group B - VAS baseline (mean ± SD): 3.58 ± 2.54 - VAS end of treatment (mean ± SD): 1.21 ± 3.15
Clinical resolution	*Clinical score using Thongprasom (score 0–5)* Group A - Baseline: Score 0: 0 Score 1: 0 Score 2: 9 (30%) Score 3: 7 (23.33%) Score 4: 12 (40%) Score 5: 2 (6.67%) - End of treatment: Score 0: 5 (21.74%) Score 1: 11 (47.82%) Score 2: 1 (4.35%) Score 3: 4 (17.39%) Score 4: 2 (8.70%) Score 5: 0 Group B - Baseline: Score 0: 0 Score 1: 0 Score 2: 10 (33.33%) Score 3: 4 (13.33%) Score 4: 14 (46.67%) Score 5: 2 (6.67%) - End of treatment: Score 0: 2 (8.70%) Score 1: 9 (39.13%) Score 2: 5 (21.73%) Score 3: 3 (13.04%) Score 4: 2 (8.70%) Score 5: 2 (8.70%) *Complete resolution (Efficacy index)* Group A: 5 patients (21.74%) Group B: 2 patients (8.70%)
Adverse effects	Group A: no adverse effects Group B: - 17 patients had minor swelling at the side treated with cryotherapy - 12 patients had pain augmentation in the first 7–10 days after cryotherapy
Follow-up	1 week after treatment
Statistical analysis	Pain improvement: both treatments significantly reduced pain in all follow-up sessions (*p* < 0.05) (Wilcoxon test); Group B showed a significantly higher improvement at week 2 (*p* = 0.038) and week 4 (*p* = 0.004), but not at week 6 (*p* = 0.690) (Paired samples *t*-test) Clinical resolution: both treatments reduced the severity of the lesions significantly in all follow-up sessions (*p* < 0.05) (Wilcoxon test) but no significant differences between the groups (Paired samples *t*-test)
Azizi 2007 [25]	
Study design	RCT
Country	Iran
Patients randomized (analyzed)	Group A: 30 (30) Group B: 30 (30)
Interventions	Group A: TA in Orabase^®^ 4 times daily for 4 weeks Group B: 0.1% TAC ointment 4 times daily for 4 weeks
Pain improvement	Group A - VAS baseline (mean): 8.2 - VAS end of treatment (mean): 3.5 Group B - VAS baseline (mean): 7.8 - VAS end of treatment (mean): 3.2
Clinical resolution	*Mean score of OLP lesions severity (score 0–5)* Group A - Baseline: 3.4 - End of treatment: 1.5 Group B - Baseline: 3.2 - End of treatment: 1.2
Adverse effects	Not reported
Follow-up	No follow-up after treatment
Statistical analysis	Pain improvement: significant changes in both groups (*p* < 0.005) (Kruskal-Wallis k-sample test) but no significant differences between the two groups Clinical resolution: no significant changes in both groups; no significant differences between the two groups following treatment
Bhatt 2022 [26]	
Study design	RCT
Country	India
Patients randomized (analyzed)	Group A: 30 (30) Group B: 30 (30)
Interventions	Group A: AV gel (obtained from 500 mg AV capsules) 3 times daily for 2 months Group B: LLLT at 980 nm twice weekly for 2 months
Pain improvement	Group A - VAS baseline (mean ± SD): 6.10 ± 0.27 - VAS end of treatment (mean ± SD): 4.47 ± 0.27 Group B - VAS baseline (mean ± SD): 6.87 ± 0.32 - VAS end of treatment (mean ± SD): 3.83 ± 0.31
Clinical resolution	*Site score (score 0–2)* Group A - Baseline (mean ± SD): 3.60 ± 0.30 - End of treatment (mean ± SD): 3.27 ± 0.31 Group B - Baseline (mean ± SD): 3.03 ± 0.22 - End of treatment (mean ± SD): 2.43 ± 0.22 *Severity score (score 0–3)* Group A - Baseline (mean ± SD): 2.30 ± 0.43 - End of treatment (mean ± SD): 1.87 ± 0.39 Group B - Baseline (mean ± SD): 2.83 ± 0.49 - End of treatment (mean ± SD): 1.77 ± 0.39 *Activity score (site score × severity score)* Group A - Baseline (mean ± SD): 3.20 ± 0.66 - End of treatment (mean ± SD): 2.37 ± 0.58 Group B - Baseline (mean ± SD): 3.80 ± 0.64 - End of treatment (mean ± SD): 1.77 ± 0.39
Adverse effects	No adverse effects
Follow-up	7 months after treatment
Statistical analysis	Pain improvement: significant changes in both groups at follow-up sessions (Mann–Whitney U test) Clinical resolution: significant changes in both groups during the treatment period (*p* < 0.05) (Mann–Whitney U test); LLLT group showed significantly better response compared to the AV group in the treatment period, but no significant differences between the groups during the follow-up
Brennan 2022 [27]	
Study design	RCT
Country	USA, Denmark, UK, Canada, Ireland, Sweden, Germany
Patients randomized (analyzed)	Group A: 33 (30) Group B: 34 (33) Group C: 40 (34) Group D: 31 (25)
Interventions	Group A: 20 μg CP patch twice daily for 4 weeks Group B: 5 μg CP patch twice daily for 4 weeks Group C: 1 μg CP patch twice daily for 4 weeks Group D: placebo (non-medicated) patch twice daily for 4 weeks
Pain improvement	Group A - VAS baseline (mean ± SD): 5.7 ± 2.44 - VAS end of treatment (mean ± SD): Group B - VAS baseline (mean ± SD): 5.9 ± 2.32 - VAS end of treatment (mean ± SD): Group C - VAS baseline (mean ± SD): 5.8 ± 2.38 - VAS end of treatment (mean ± SD): Group D - VAS baseline (mean ± SD): 5.8 ± 2.22 - VAS end of treatment (mean ± SD):
Clinical resolution	*Total ulcer area per patient (mm^2^)* Group A: mean change from baseline: −43.8 Group B: mean change from baseline: −49.7 Group C: mean change from baseline: −18.1 Group D: mean change from baseline: 1.3 *Total lesion area per patient (mm^2^)* Group A: mean change from baseline: −293.1 Group B: mean change from baseline: −183.6 Group C: mean change from baseline: −157.3 Group D: mean change from baseline: −124.4 *Average erythema severity score (score 0–4)* Group A: mean change from baseline: −1.182 Group B: mean change from baseline: −0.987 Group C: mean change from baseline: −0.829 Group D: mean change from baseline: −0.957 *Disease activity score (DAS) (score 0–72)* Group A: mean change from baseline: −3.450 Group B: mean change from baseline: −1.710 Group C: mean change from baseline: −1.680 Group D: mean change from baseline: −1.234 *Clinical global impression (CGI) (score 0–4)* Group A: mean change from baseline: −0.986 Group B: mean change from baseline: −0.765 Group C: mean change from baseline: −0.718 Group D: mean change from baseline: −0.848
Adverse effects	Group A: few patients reported nausea, local pain, facial pain, hemorrhage, headache Group B: few patients reported candidiasis, gingival pain and bleeding, salivary hypersecretion, local hypersensitivity, site injury Group C: few patients reported site infections, candidiasis, nausea, gingival bleeding, pain, stomatitis, hemorrhage, dysgeusia Group D: few patients reported site infections, candidiasis, diarrhea, sleep disorder, salivary hypersecretion
Follow-up	2 weeks after treatment
Statistical analysis	Pain improvement and clinical resolution: statistically significant improvement for 20-μg group in ulcer area (*p* = 0.047), symptom severity (*p* = 0.001), disease activity (*p* = 0.022), pain (*p* = 0.012), and quality of life (*p* = 0.003) as compared with placebo (closed testing procedure).
Hettiarachchi 2017 [28]	
Study design	RCT
Country	Sri Lanka
Patients randomized (analyzed)	Group A: 34 (34) Group B: 34 (34)
Interventions	Group A: 0.05% CP cream twice daily for 3 weeks + 5 mL nystatin suspension (100,000 units/mL) mouth rinse twice daily for 3 weeks Group B: 0.1% TAC cream twice daily for 3 weeks + 5 mL nystatin suspension (100,000 units/mL) mouth rinse twice daily for 3 weeks
Pain improvement	Group A - VAS baseline (mean ± SD): 1.56 ± 0.79 (right side), 1.44 ± 0.66 (left side) - VAS end of treatment (mean ± SD): 0.79 ± 0.95 (right side), 0.74 ± 0.75 (left side) Group B - VAS baseline (mean ± SD): 1.91 ± 0.87 (right side), 1.85 ± 0.78 (left side) - VAS end of treatment (mean ± SD): 0.71 ± 0.76 (right side), 0.76 ± 0.82 (left side)
Clinical resolution	*Clinical score using Thongprasom (score 0–5)* Group A - Baseline (mean ± SD): 2.00 ± 1.10 (right side), 2.00 ± 1.10 (left side) - End of treatment (mean ± SD): 1.79 ± 0.95 (right side), 1.79 ± 0.84 (left side) Group B - Baseline (mean ± SD): 2.71 ± 1.40 (right side), 2.56 ± 1.31 (left side) - End of treatment (mean ± SD): 1.88 ± 0.91 (right side), 1.94 ± 0.89 (left side)
Adverse effects	No adverse effects
Follow-up	2 weeks after treatment
Statistical analysis	Pain improvement: significant changes (*p* < 0.05) in both groups for both sides (Pearson’s χ^2^-test) Clinical resolution: significant changes (*p* < 0.05) in mean clinical scores in both groups for both sides (Pearson’s χ^2^-test); TAC group was significantly more effective than clobetasol group (*p* < 0.05) (Student’s *t*-test)
Kazancioglu 2015 [29]	
Study design	RCT
Country	Turkey
Patients randomized (analyzed)	Group A: 30 (30) Group B: 30 (30) Group C: 30 (30) Group D: 30 (30)
Interventions	Group A: LLLT with diode laser (808 nm, 0.1 W) irradiation (exposure time 2.5 min; fluence 1.5 J/cm^2^ per session; irradiance 10 mW/cm^2^; area 1 cm^2^), twice a week (once every third day) for a maximum of 10 sessions Group B: ozone generator intraorally with an intensity of 60% for 10 s, concentration of ozone in the operation field of 10–100 μg/mL, twice a week (once every third day) for a maximum of 10 sessions Group C: dexamethasone mouth rinse for 5 min + 30 min later mouth rinse with 30 drops of nystatin (100,000 units) for 5 min, 4 times daily for 1 month Group D (placebo): ointment without the active corticosteroid gargled for 5 min, 4 times a day for 1 month
Pain improvement	Group A - VAS baseline (mean ± SD): 4.10 ± 1.80 - VAS end of treatment (mean ± SD): 0.33 ± 1.60 Group B - VAS baseline (mean ± SD): 5.01 ± 1.20 - VAS end of treatment (mean ± SD): 0.25 ± 1.20 Group C - VAS baseline (mean ± SD): 5.00 ± 0.90 - VAS end of treatment (mean ± SD): 0.22 ± 1.00 Group D - VAS baseline (mean ± SD): 4.38 ± 1.10 - VAS end of treatment (mean ± SD): 4.03 ± 1.90
Clinical resolution	*Mean sign score (0–5)* Group A - Baseline: 3.75 - End of treatment: 2.50 Group B - Baseline: 3.80 - End of treatment: 1.80 Group C - Baseline: 3.85 - End of treatment: 1.60 Group D - Baseline: 3.80 - End of treatment: 3.35 *Clinical severity of lesion* Group A - Baseline (mean ± SD): 7.23 ± 3.6 - End of treatment (mean ± SD): 3.01 ± 1.1 Group B - Baseline (mean ± SD): 7.51 ± 4.1 - End of treatment (mean ± SD): 2.66 ± 1.5 Group C - Baseline (mean ± SD): 7.41 ± 3.1 - End of treatment (mean ± SD): 2.43 ± 1.0 Group D - Baseline (mean ± SD): 7.44 ± 5.7 - End of treatment (mean ± SD): 7.34 ± 1.5 *Complete resolution (Efficacy index)* Group A: 0 patients (0%) Group B: 3 patients (10%) Group C: 6 patients (20%) Group D: 0 patients (0%)
Adverse effects	No adverse effects
Follow-up	3 months after treatment
Statistical analysis	Pain improvement: significant changes in group A, B, and C (*p* < 0.05) (Wilcoxon sign test) but no significant differences between the groups after the treatment (Paired *t*-test) Clinical resolution: significant improvements in group B and C (*p* < 0.05) (Mann–Whitney U-test) but no significant differences between the groups (Paired *t*-test)
Liu 2013 [30]	
Study design	RCT
Country	West China
Patients randomized (analyzed)	Group A: 30 (29) Group B: 31 (30)
Interventions	Group A: intralesional injection of 1.4 mg betamethasone, once weekly for 2 weeks Group B: intralesional injection of 8 mg TA (40 mg/mL), once weekly for 2 weeks
Pain improvement	Group A: VAS reduction at 14 ± 2 days (mean ± SD): 3.41 ± 2.03 Group B: VAS reduction at 14 ± 2 days (mean ± SD): 3.00 ± 2.13
Clinical resolution	*Reduction in erosive area (mm^2^)* Group A: 14 ± 2 days (mean ± SD): 21.28 ± 21.06 Group B: 14 ± 2 days (mean ± SD): 11.50 ± 12.9
Adverse effects	Group A: 1 patient had slight burning sensation in the throat Group B: no adverse effects
Follow-up	3 months after treatment
Statistical analysis	Pain improvement: no significant differences between the groups (Wilcoxon rank-sum test) Clinical resolution: significantly greater reduction in erosive area in group A compared to group B (Wilcoxon rank-sum test)
Lopez Jornet 2016 [31]	
Study design	RCT
Country	Spain
Patients randomized (analyzed)	Group A: 30 (26) Group B: 30 (29)
Interventions	Group A: 2% Chamaemelum Nobile gel, 0.5 mL 3 times daily for 1 month Group B: placebo gel, 0.5 mL 3 times daily for 1 month
Pain improvement	Group A - VAS baseline (mean ± SD): 5.15 ± 1.70 - VAS end of treatment (mean ± SD): 1.88 ± 1.30 Group B - VAS baseline (mean ± SD): 4.21 ± 1.80 - VAS end of treatment (mean ± SD): 4.31 ± 1.90
Clinical resolution	*Clinical score using Thongprasom (score 0–5)* Group A - Baseline (mean ± SD): 2.08 ± 0.90 - End of treatment (mean ± SD): 1.09 ± 0.50 Group B - Baseline (mean ± SD): 1.72 ± 0.90 - End of treatment (mean ± SD): 1.64 ± 0.70 *Complete resolution (Efficacy index)* Group A: 5 patients (19.23%) Group B: 0 patients (0%)
Adverse effects	No adverse effects
Follow-up	No follow-up after treatment
Statistical analysis	Pain improvement: significant changes in group A after treatment (*p* < 0.001) (ANOVA test) Clinical resolution: significant changes in group A after treatment (*p* < 0.001) (ANOVA test)
Manjunatha 2012 [32]	
Study design	RCT
Country	India
Patients randomized (analyzed)	Group A: 30 (30) Group B: 30 (30)
Interventions	Group A: 0.1% TAC in Orabase^®^, 3 times daily for 2 weeks Group B: 0.1% TA in Orabase^®^, 3 times daily for 2 weeks
Pain improvement	Group A - VAS baseline (mean ± SD): 7.80 ± 1.90 - VAS end of treatment (mean ± SD): 1.20 ± 2.60 Group B - VAS baseline (mean ± SD): 7.60 ± 2.01 - VAS end of treatment (mean ± SD): 2.70 ± 2.50
Clinical resolution	*Clinical score using Thongprasom (score 0–5)* Group A - Baseline (mean ± SD): 3.10 ± 1.27 - End of treatment (mean ± SD): 2.30 ± 1.10 Group B - Baseline (mean ± SD): 2.73 ± 1.46 - End of treatment (mean ± SD): 1.20 ± 1.40 *Complete resolution (Efficacy index)* Group A: 19 patients (63.33%) Group B: 2 patients (6.67%)
Adverse effects	No adverse effects
Follow-up	2 weeks after treatment
Statistical analysis	Pain improvement: significantly better response at day 21 (*p* = 0.0309) and 28 (*p* = 0.0001) for group A compared to group B (Student paired *t*-test/Wilson-Wilcoxon matched paired test). Clinical resolution: significantly better response for group A (*p* = 0.002) compared to group B (Student paired *t*-test/Wilson-Wilcoxon matched paired test).
Salazar-Sanchez 2010 [33]	
Study design	RCT
Country	Spain
Patients randomized (analyzed)	Group A: 32 (31) Group B: 32 (24)
Interventions	Group A: 70% AV gel (0.4 mL), 3 times daily for 12 weeks Group B: placebo gel, 3 times daily for 12 weeks
Pain improvement	Group A - VAS baseline (mean ± SD): 5.50 ± 2.10 - VAS end of treatment (mean ± SD): 2.50 ± 3.00 Group B - VAS baseline (mean ± SD): 5.80 ± 1.80 - VAS end of treatment (mean ± SD): 3.70 ± 3.30
Clinical resolution	*Clinical score using Thongprasom (score 0–5)* Group A - Baseline (mean ± SD): 3.03 ± 0.98 - End of treatment (mean ± SD): 1.74 ± 1.26 Group B - Baseline (mean ± SD): 3.00 ± 1.28 - End of treatment (mean ± SD): 1.83 ± 1.16 *Complete resolution (Efficacy index)* Group A: 19 patients (61.29%) Group B: 10 patients (32.26%)
Adverse effects	No adverse effects
Follow-up	No follow-up after treatment
Statistical analysis	Pain improvement: no significant differences between groups after 6 (*p* = 0.508) and 12 weeks (*p* = 0.345) of treatment (Student’s *t*-test) Clinical resolution: no significant differences between groups after 6 (*p* = 0.082) and 12 weeks (*p* = 0.344) of treatment (Pearson’s chi-squared test)
Scardina 2006 [34]	
Study design	RCT
Country	Italy
Patients randomized (analyzed)	Group A: 35 (35) Group B: 35 (35)
Interventions	Group A: isotretinoin 0.18% twice daily for 3 months Group B: isotretinoin 0.05% twice daily for 3 months
Pain improvement	Group A - VAS baseline (mean): 7.7 - VAS end of treatment (mean): 0 Group B - VAS baseline (mean): 7.8 - VAS end of treatment (mean): 4.2
Clinical resolution	*Clinical score (score 0–3)* Group A - Baseline (mean): 2.4 - End of treatment (mean): 1.3 Group B - Baseline (mean): 2.5 - End of treatment (mean): 2.1
Adverse effects	Group A and B: few patients had a transitory increase in soreness and pain for 30 min after treatment
Follow-up	A group of 20 patients had a 5-year follow-up, and a group of 20 patients had a 10-year follow-up
Statistical analysis	Pain improvement: prior to therapy, the mean VAS in group A and group B was 7.7 and 7.8, respectively (*p* > 0.01). At the end of therapy, the mean VAS was 0 and 4.2 in group A and group B, respectively (*p* < 0.01) Clinical resolution: significantly greater changes in patients of group A compared to group B (*p* < 0.01) (Wilcoxon rank sum test)
Wu 2010 [35]	
Study design	RCT
Country	China
Patients randomized (analyzed)	Group A: 37 (33) Group B: 32 (30)
Interventions	Group A: thalidomide paste 1% (galenic), 3 times daily for 1 week Group B: dexamethasone paste 0.043% (galenic), 3 times daily for 1 week
Pain improvement	Group A - VAS baseline (mean ± SD): 4.17 ± 2.19 - VAS end of treatment (mean ± SD): 1.40 ± 1.54 Group B - VAS baseline (mean ± SD): 3.79 ± 1.51 - VAS end of treatment (mean ± SD): 0.91 ± 1.28
Clinical resolution	*Size of erosive area (mm^2^)* Group A - Baseline (mean ± SD): 38.94 ± 49.66 - End of treatment (mean ± SD): 6.47 ± 16.00 Group B - Baseline (mean ± SD): 31.13 ± 36.49 - End of treatment (mean ± SD): 7.82 ± 20.06 *Complete resolution (Efficacy index)* Group A: 18 patients (54.55%) Group B: 17 patients (56.67%)
Adverse effects	Group A: 2 patients had burning and tingling sensations Group B: 2 patients had burning and tingling sensations
Follow-up	1-month and 3-month follow-up to detect recurrences. 1-year follow-up to detect adverse reactions
Statistical analysis	Pain improvement: significant reductions in VAS scores in both groups compared to baseline (*p* < 0.001) (Wilcoxon test) but no significant differences between groups (*p* = 0.498) after treatment (Mann-Whitney U-test) Clinical resolution: significant improvements in both groups (*p* < 0.001) (Wilcoxon test) but no significant differences between groups after treatment (*p* = 0.420) (Mann-Whitney U-test)
Xia 2006 [36]	
Study design	Split-mouth RCT
Country	China
Patients randomized (analyzed)	Group A: 45 (right buccal mucosa) Group B: 45 (left buccal mucosa)
Interventions	Group A: 0.5 mL intralesional injection of TA (40 mg/mL) Group B: no treatment
Pain improvement	Group A - VAS baseline (mean ± SD): 52.42 ± 11.72 - VAS end of treatment (mean ± SD): 8.33 ± 10.11 Group B - VAS baseline (mean ± SD): 51.36 ± 13.00 - VAS end of treatment (mean ± SD): 49.69 ± 12.85
Clinical resolution	*Size of erythematous lesions (mm^2^)* Group A - Baseline (mean ± SD): 155.78 ± 32.65 - End of treatment (mean ± SD): 32.82 ± 18.00 Group B - Baseline (mean ± SD): 147.02 ± 28.65 - End of treatment (mean ± SD): 145.49 ± 29.05 *Size of ulcerative lesions (mm^2^)* Group A - Baseline (mean ± SD): 34.27 ± 24.29 - End of treatment (mean ± SD): 7.64 ± 8.63 Group B - Baseline (mean ± SD): 33.47 ± 21.95 - End of treatment (mean ± SD): 32.27 ± 19.90 *REU score* Group A - Baseline (mean ± SD): 6.01 ± 0.38 - End of treatment (mean ± SD): 3.83 ± 0.95 Group B - Baseline (mean ± SD): 5.94 ± 0.49 - End of treatment (mean ± SD): 5.94 ± 0.49
Adverse effects	No adverse effects
Follow-up	2 weeks after treatment
Statistical analysis	Pain improvement and clinical resolution: both symptoms and signs were significantly reduced in group A (*p* < 0.05) when comparing the 1-week follow-up to the baseline and the 2-week follow-up to the 1-week follow-up (ANOVA). No changes were observed in the control group (*p* > 0.05)
Yoke 2006 [37]	
Study design	RCT
Country	Singapore, South Korea, India, Thailand
Patients randomized (analyzed)	Group A: 68 (66) Group B: 71 (71)
Interventions	Group A: cyclosporin 0.1% solution thrice daily for 8 weeks Group B: 0.1% TA in Orabase^®^ 3 times daily for 8 weeks
Pain improvement	Group A - VAS baseline (mean ± SD): 22.88 ± 27.32 - VAS end of treatment (mean ± SD): 15.56 ± 24.42 Group B - VAS baseline (mean ± SD): 23.72 ± 24.53 - VAS end of treatment (mean ± SD): 13.29 ± 23.35
Clinical resolution	*Clinical score using Thongprasom (score 0–5)* Group A - Baseline (mean ± SD): 2.82 ± 1.31 - End of treatment (mean ± SD): not reported Group B - Baseline (mean ± SD): 2.56 ± 1.39 - End of treatment (mean ± SD): not reported *Size of reticulation (mm^2^)* Group A - Baseline (mean ± SD): 175.55 ± 242.51 - End of treatment (mean ± SD): 90.36 ± 99.22 Group B - Baseline (mean ± SD): 141.72 ± 117.88 - End of treatment (mean ± SD): 48.95 ± 60.73 *Size of erythematous lesions (mm^2^)* Group A - Baseline (mean ± SD): 122.80 ± 105.11 - End of treatment (mean ± SD): 66.70 ± 107.37 Group B - Baseline (mean ± SD): 91.65 ± 90.16 - End of treatment (mean ± SD): 40.08 ± 73.03 *Size of ulcerative lesions (mm^2^)* Group A - Baseline (mean ± SD): 33.39 ± 54.45 - End of treatment (mean ± SD): 20.67 ± 39.69 Group B - Baseline (mean ± SD): 20.81 ± 22.76 - End of treatment (mean ± SD): 4.91 ± 16.11 *Clinical response (after 8 weeks)* Group A: 29/66 patients (44%) Group B: 41/71 patients (58%)
Adverse effects	Group A - 14 patients had transient burning sensation - 1 patient had local swelling and itching - 4 patients had gastrointestinal upsets Group B: 3 patients had transient burning sensation
Follow-up	Follow-up assessments after the treatment period were at week 12 and then every 3 months for 1 year
Statistical analysis	Pain improvement and clinical resolution: no statistically significant differences between the groups regarding all parameters (two tailed *t*-Test)
Zborowski 2021 [38]	
Study design	Split-mouth RCT
Country	Poland
Patients randomized (analyzed)	Group A: 30 (28) Group B: 30 (28)
Interventions	Group A: MB-PDT in 4 sessions on days 1, 3, 6, 9, with a diode laser (spot size: 0.8 cm^2^ at 650 nm; energy fluence: 120 J/cm^2^; power density: 1034 mW/cm^2^ for 227 s) + on days 2, 4, 5, 7, 8 of the treatment, a carrier adapted to the size of the lesion was self-administered by the patient Group B: 0.05% TA on days 1, 3, 6, 9 + on days 2, 4, 5, 7, 8 of the treatment, a carrier adapted to the size of the lesion was self-administered by the patient
Pain improvement	Group A - VAS baseline (mean ± SD): 4.86 ± 2.70 - VAS end of treatment (mean ± SD): 1.96 ± 2.50 Group B - VAS baseline (mean ± SD): 4.86 ± 2.70 - VAS end of treatment (mean ± SD): 1.96 ± 2.50
Clinical resolution	*Size of lesions (cm^2^)* Group A - Baseline (mean ± SD): 17.83 ± 16.60 - After treatment (mean ± SD): 10.71 ± 11.70 Group B - Baseline (mean ± SD): 15.53 ± 13.20 - After treatment (mean ± SD): 9.12 ± 8.40 *Clinical score using Thongprasom (score 0–5)* Group A - Baseline (mean ± SD): 2.87 ± 0.70 - After treatment (mean ± SD): 2.13 ± 1.00 Group B - Baseline (mean ± SD): 3.15 ± 0.60 - After treatment (mean ± SD): 2.41 ± 1.00 *Treatment efficacy* Group A - Complete response: 8/24 patients (33.33%) - Partial response: 10/24 patients (41.67%) No response: 6/24 patients (25%) Group B - Complete response: 6/27 patients (22.22%) - Partial response: 18/27 patients (66.67%) - No response: 3/27 patients (11.11%)
Adverse effects	Group A: 4 patients had slight swelling and increased pain after the first or second PDT session Group B: - 1 patient abandoned self-administration of TA because of technical difficulties with carrier application - 1 patient had halitosis
Follow-up	3 months after treatment
Statistical analysis	Pain improvement: significant changes in both groups immediately after treatment and three months later (Wilcoxon paired rank test) but no significant differences between the groups Clinical resolution: lesions’ size and clinical score significantly decreased in both groups after treatment and during follow-up (Wilcoxon paired rank test) but no significant differences between the groups
