# Supplementary material for: Efficacy of Topical Treatments for the Management of Symptomatic Oral Lichen Planus: A Systematic Review
Source: Int J Environ Res Public Health. 2023 Jan 10;20(2):1202. doi: 10.3390/ijerph20021202 (PMC9859481; doi:10.3390/ijerph20021202)
Supplement: Supplementary file 1 [file ijerph-20-01202-s001.zip › ijerph-2054763-supplementary.pdf]

**Table S1**. Characteristics of excluded studies.

| Authors | Year | Title | Reason for exclusion |
|---|---|---|---|
| Arduino PG, Campolongo MG, Sciannameo V, Conrotto D, Gambino A, Cabras M, Ricceri F, Carossa S, Broccoletti R, Carbone M | 2018 | Randomized, placebo-controlled, double-blind trial of clobetasol propionate 0.05% in the treatment of oral lichen planus | Inadequate sample size |
| Arunkumar S, Kalappanavar AN, Annigeri NG, Kalappa SG | 2015 | Relative efficacy of pimecrolimus cream and triamcinolone acetonide paste in the treatment of symptomatic oral lichen planus | Inadequate sample size |
| Azizi A, Dadras OG, Jafari M, Ghadim NM, Lawaf S, Sadri D | 2015 | Efficacy of 0.1% triamcinolone with nanoliposomal carrier formulation in orabase for oral lichen planus patients: a clinical trial | No randomization (quasi-randomized trial) |
| Bacci C, Vanzo V, Frigo AC, Stellini E, Sbricoli L, Valente M | 2017 | Topical tocopherol for treatment of reticular oral lichen planus: a randomized, double-blind, crossover study | Inadequate sample size and insufficient data to assess outcomes |
| Bakhtiari S, Azari-Marhabi S, Mojahedi SM, Namdari M, Rankohi ZE, Jafari S | 2017 | Comparing clinical effects of photodynamic therapy as a novel method with topical corticosteroid for treatment of oral lichen planus | Inadequate sample size |
| Belal MH | 2015 | Management of symptomatic erosive-ulcerative lesions of oral lichen planus in an adult Egyptian population using Selenium-ACE combined with topical corticosteroids plus antifungal agent | Inadequate sample size and insufficient data to assess outcomes |
| Bendas ER, Abdullah H, El-Komy MH, Kassem MA | 2013 | Hydroxychloroquine niosomes: a new trend in topical management of oral lichen planus | Inadequate sample size and insufficient data to assess outcomes |
| Bennardo F, Liborio F, Barone S, Antonelli A, Buffone C, Fortunato L, Giudice A | 2021 | Efficacy of platelet-rich fibrin compared with triamcinolone acetonide as injective therapy in the treatment of symptomatic oral lichen planus: a pilot study | Inadequate sample size |
| Carbone M, Arduino PG, Carrozzo M, Caiazzo G, Broccoletti R, Conrotto D, Bezzo C, Gandolfo S | 2009 | Topical clobetasol in the treatment of atrophic-erosive oral lichen planus: a randomized controlled trial to compare two preparations with different concentrations | Inadequate sample size |
| Chaitanya NC, Chikte D, Kumar YP, Komali G, Yellarthi SP, Reddy CS, Harika DP, Haritha S, Taie WAA, Hatab NA, Patil S, Panta P | 2022 | Efficacy of spirulina 500 mg vs triamcinolone acetonide 0.1% for the treatment of oral lichen planus: a randomized clinical trial | Associated systemic therapy |
| Choonhakarn C, Busaracome P, Sripanidkulchai B, Sarakarn P | 2008 | The efficacy of aloe vera gel in the treatment of oral lichen planus: a randomized controlled trial | Inadequate sample size |
| Cilurzo F, Gennari CG, Selmin F, Epstein JB, Gaeta GM, Colella G, Minghetti P | 2010 | A new mucoadhesive dosage form for the management of oral lichen planus: formulation study and clinical study | Included patients without symptoms |
| Conrotto D, Carbone M, Carrozzo M, Arduino P, Broccoletti R, Pentenero M, Gandolfo S | 2006 | Ciclosporin vs. clobetasol in the topical management of atrophic and erosive oral lichen planus: a double-blind, randomized controlled trial | Inadequate sample size |
| Corrocher G, Di Lorenzo G, Martinelli N, Mansueto P, Biasi D, Nocini PF, Lombardo G, Fior A, Corrocher R, Bambara LM, Gelio S, Pacor ML | 2008 | Comparative effect of tacrolimus 0.1% ointment and clobetasol 0.05% ointment in patients with oral lichen planus | Inadequate sample size and insufficient data to assess outcomes |
| Dalirsani Z, Taghavi Zenouz A, Mehdipour M, Alavi F, Javadzadeh Y | 2010 | Comparison of the effect of combination of triamcinolone acetonide and vitamin a mouthwash with triamcinolone mouthwash alone on oral lichen planus | Inadequate sample size and insufficient data to assess outcomes |
| Dillenburg CS, Martins MA, Munerato MC, Marques MM, Carrard VC, Sant'Ana Filho M, Castilho RM, Martins MD | 2014 | Efficacy of laser phototherapy in comparison to topical clobetasol for the treatment of oral lichen planus: a randomized controlled trial | Inadequate sample size |
| El Shenawy HM, Eldin AM | 2015 | A comparative evaluation of low-level laser and topical steroid therapies for the treatment of erosive-atrophic lichen planus | No randomization (quasi-randomized trial) |

| | | | |
|---|---|---|---|
| Ezzatt OM, Helmy IM | 2019 | Topical pimecrolimus versus betamethasone for oral lichen planus: a randomized clinical trial | Inadequate sample size |
| Ferri EP, Gallo CB, Abboud CS, Yanaguizawa WH, Horliana ACRT, Silva DFTD, Pavani C, Bussadori SK, Nunes FD, Mesquita-Ferrari RA, Fernandes KPS, Rodrigues MFSD | 2018 | Efficacy of photobiomodulation on oral lichen planus: a protocol study for a double-blind, randomised controlled clinical trial | Protocol study |
| Ferri EP, Cunha KRL, Abboud CS, de Barros Gallo C, de Sousa Sobral S, de Fatima Teixeira da Silva D, Horliana ACRT, Franco AL, Rodrigues MFSD | 2021 | Photobiomodulation is effective in oral lichen planus: a randomized, controlled, double-blind study | Inadequate sample size |
| Fu J, Zhu X, Dan H, Zhou Y, Liu C, Wang F, Li Y, Liu N, Chen Q, Xu Y, Zeng X, Jiang L | 2012 | Amlexanox is as effective as dexamethasone in topical treatment of erosive oral lichen planus: a short-term pilot study | Inadequate sample size and insufficient data to assess outcomes |
| Georgaki M, Piperi E, Theofilou VI, Pettas E, Stoufi E, Nikitakis NG | 2022 | A randomized clinical trial of topical dexamethasone vs. cyclosporine treatment for oral lichen planus | Inadequate sample size |
| Gorouhi F, Solhpour A, Beitollahi JM, Afshar S, Davari P, Hashemi P, Nassiri Kashani M, Firooz A | 2007 | Randomized trial of pimecrolimus cream versus triamcinolone acetonide paste in the treatment of oral lichen planus | Inadequate sample size |
| Hambly JL, Haywood A, Hattingh L, Nair RG | 2017 | Comparison between self-formulation and compounded-formulation dexamethasone mouth rinse for oral lichen planus: a pilot, randomized, cross-over trial | No randomization (quasi-randomized trial) |
| Hashem AS, Issrani R, Elsayed TEE, Prabhu N | 2019 | Topical hyaluronic acid in the management of oral lichen planus: a comparative study | No data on clinical improvement |
| Hesen RR, Abuel-Ela HA, Helmy IM, El Sayed MH, Ezzatt OM | 2017 | Glucosamine as a novel adjunctive therapy in symptomatic oral lichen planus | Inadequate sample size and insufficient data to assess outcomes |
| Hijazi A, Ahmed W, Gaafar S | 2022 | Efficacy of intralesional injections of platelet-rich plasma in patients with oral lichen planus: a pilot randomized clinical trial | Inadequate sample size and insufficient data to assess outcomes |
| Jajarm HH, Falaki F, Mahdavi O | 2011 | A comparative pilot study of low intensity laser versus topical corticosteroids in the treatment of erosive-atrophic oral lichen planus | No randomization (quasi-randomized trial) |
| Jajarm HH, Falaki F, Sanatkhani M, Ahmadzadeh M, Ahrari F, Shafaee H | 2015 | A comparative study of toluidine blue-mediated photodynamic therapy versus topical corticosteroids in the treatment of erosive-atrophic oral lichen planus: a randomized clinical controlled trial | Inadequate sample size |
| Joshy A, Doggalli N, Patil K, Kulkarni PK | 2018 | To evaluate the efficacy of topical propolis in the management of symptomatic oral lichen planus: a randomized controlled trial | Inadequate sample size and insufficient data to assess outcomes |
| Kia SJ, Shirazian S, Mansourian A, Khodadadi Fard L, Ashnagar S | 2015 | Comparative efficacy of topical curcumin and triamcinolone for oral lichen planus: a randomized, controlled clinical trial | Inadequate sample size |
| Laeijendecker R, Tank B, Dekker SK, Neumann HA | 2006 | A comparison of treatment of oral lichen planus with topical tacrolimus and triamcinolone acetonide ointment | Insufficient data to assess outcomes |
| Lavaee F, Shadmanpour M | 2019 | Comparison of the effect of photodynamic therapy and topical corticosteroid on oral lichen planus lesions | Inadequate sample size |
| Lee YC, Shin SY, Kim SW, Eun YG | 2013 | Intralesional injection versus mouth rinse of triamcinolone acetonide in oral lichen planus: a randomized controlled study | Included patients without symptoms |
| Li Y, Shao F, Zheng S, Tan Z, He Y | 2020 | Alteration of Streptococcus salivarius in buccal mucosa of oral lichen planus and controlled clinical trial in OLP treatment | Inadequate sample size |
| Lodi G, Tarozzi M, Sardella A, Demarosi F, Canegallo L, Di Benedetto D, Carrassi A | 2007 | Miconazole as adjuvant therapy for oral lichen planus: a double-blind randomized controlled trial | Inadequate sample size and insufficient data on clinical improvement |
| Malhotra AK, Khaitan BK, Sethuraman G, Sharma VK | 2008 | Betamethasone oral mini-pulse therapy compared with topical triamcinolone acetonide (0.1%) paste in oral lichen planus: a randomized comparative study | Inadequate sample size and insufficient data on pain improvement |
| Mamadapur R, Naik Z, Kumar SL, Bagewadi A | 2022 | Comparative efficacy of topical coconut cream and clobetasol propionate ointment for the management of oral lichen planus: a double-blinded randomized control trial | Insufficient data on clinical improvement |

| | | | |
|---|---|---|---|
| Mansourian A, Momen-Heravi F, Saheb-Jamee M, Esfehani M, Khalilzadeh O, Momen-Beitollahi J | 2011 | Comparison of aloe vera mouthwash with triamcinolone acetonide 0.1% on oral lichen planus: a randomized double-blinded clinical trial | Inadequate sample size |
| McCaughey C, Machan M, Bennett R, Zone JJ, Hull CM | 2011 | Pimecrolimus 1% cream for oral erosive lichen planus: a 6-week randomized, double-blind, vehicle-controlled study with a 6-week open-label extension to assess efficacy and safety | Included patients without symptoms |
| Mehdipour M, Taghavi Zenouz A, Bahramian A, Yazdani J, Pouralibaba F, Sadr K | 2010 | Comparison of the effect of mouthwashes with and without zinc and fluocinolone on the healing process of erosive oral lichen planus | Inadequate sample size and insufficient data to assess outcomes |
| Mirza S, Rehman N, Alrahlah A, Alamri WR, Vohra F | 2018 | Efficacy of photodynamic therapy or low level laser therapy against steroid therapy in the treatment of erosive-atrophic oral lichen planus | Inadequate sample size |
| Mostafa D, Moussa E, Alnouaem M | 2017 | Evaluation of photodynamic therapy in treatment of oral erosive lichen planus in comparison with topically applied corticosteroids | Inadequate sample size |
| Mostafa B, Zakaria M | 2018 | Evaluation of combined topical ozone and steroid therapy in management of oral lichen planus | Included patients without symptoms |
| Nolan A, Badminton J, Maguire J, Seymour RA | 2009 | The efficacy of topical hyaluronic acid in the management of oral lichen planus | Insufficient data to assess outcomes |
| Nosratzehi T, Arbabi-Kalati F, Hamishehkar H, Bagheri S | 2018 | Comparison of the effects of curcumin mucoadhesive paste and local corticosteroid on the treatment of erosive oral lichen planus lesions | Inadequate sample size |
| Pakfetrat A, Delavarian Z, Falaki F, Khorashadizadeh M, Saba M | 2015 | The effect of pimecrolimus cream 1% compared with triamcinolone acetonide paste in treatment of atrophic-erosive oral lichen planus | Inadequate sample size |
| Passeron T, Lacour JP, Fontas E, Ortonne JP | 2007 | Treatment of oral erosive lichen planus with 1% pimecrolimus cream: a double-blind, randomized, prospective trial with measurement of pimecrolimus levels in the blood | Inadequate sample size and insufficient data to assess outcomes |
| Qataya PO, Elsayed NM, Elguindy NM, Ahmed Hafiz M, Samy WM | 2020 | Selenium: A sole treatment for erosive oral lichen planus (randomized controlled clinical trial) | Inadequate sample size |
| Radfar L, Wild RC, Suresh L | 2008 | A comparative treatment study of topical tacrolimus and clobetasol in oral lichen planus | Inadequate sample size |
| Reddy RL, Reddy RS, Ramesh T, Singh TR, Swapna LA, Laxmi NV | 2012 | Randomized trial of aloe vera gel vs triamcinolone acetonide ointment in the treatment of oral lichen planus | Inadequate sample size |
| Rogulj AA, Z Alajbeg I, Brailo V, Škrinjar I, Žužul I, Vučićević-Boras V, Alajbeg I | 2021 | Topical NAVS naphthalan for the treatment of oral lichen planus and recurrent aphthous stomatitis: a double blind, randomized, parallel group study | Inadequate sample size and insufficient data on clinical resolution |
| Saglam E, Ozsagir ZB, Unver T, Alinca SB, Toprak A, Tunali M | 2021 | Efficacy of injectable platelet-rich fibrin in the erosive oral lichen planus: a split-mouth, randomized, controlled clinical trial | Inadequate sample size |
| Saleh W, Tageldin S, Khashaba E, Darwish M, Elnagdy S, Khashaba O | 2020 | Could photodynamic therapy be utilized as a treatment modality for oral lichen planus? | Inadequate sample size and insufficient data to assess outcomes |
| Samiee N, Taghavi Zenuz A, Mehdipour M, Shokri J | 2020 | Treatment of oral lichen planus with mucoadhesive mycophenolate mofetil patch: a randomized clinical trial | Inadequate sample size and insufficient data to assess outcomes |
| Samimi M, Le Gouge A, Boralevi F, Passeron T, Pascal F, Bernard P, Agbo-Godeau S, Leducq S, Fricain JC, Vaillant L, Francès C | 2020 | Topical rapamycin versus betamethasone dipropionate ointment for treating oral erosive lichen planus: a randomized, double-blind, controlled study | Insufficient data on clinical improvement |
| Santonocito S, Polizzi A, De Pasquale R, Ronsivalle V, Lo Giudice A, Isola G | 2020 | Analysis of the efficacy of two treatment protocols for patients with symptomatic oral lichen planus: a randomized clinical trial | Inadequate sample size |
| Shetty RR, Burde KN, Guttal KS | 2016 | The efficacy of topical hyaluronic acid 0.2% in the management of symptomatic oral lichen planus | Inadequate sample size |
| Singh AR, Rai A, Aftab M, Jain S, Singh M | 2017 | Efficacy of steroidal vs non-steroidal agents in oral lichen planus: a randomised, open-label study | Inadequate sample size |
| Siponen M, Huuskonen L, Kallio-Pulkkinen S, Nieminen P, Salo T | 2017 | Topical tacrolimus, triamcinolone acetonide, and placebo in oral lichen planus: a pilot randomized controlled trial | Inadequate sample size |
| Sivaraman S, Santham K, Nelson A, Laliytha B, Azhalvel P, Deepak JH | 2016 | A randomized triple-blind clinical trial to compare the effectiveness of topical triamcinolone acetonate (0.1%), | Inadequate sample size |

| | | | |
|---|---|---|---|
| | | clobetasol propionate (0.05%), and tacrolimus orabase (0.03%) in the management of oral lichen planus | |
| Sonthalia S, Singal A | 2012 | Comparative efficacy of tacrolimus 0.1% ointment and clobetasol propionate 0.05% ointment in oral lichen planus: a randomized double-blind trial | Inadequate sample size |
| Swift JC, Rees TD, Plemons JM, Hallmon WW, Wright JC | 2005 | The effectiveness of 1% pimecrolimus cream in the treatment of oral erosive lichen planus | Inadequate sample size |
| Beigom Taheri J, Anbari F, Maleki Z, Boostani S, Zarghi A, Pouralibaba F | 2010 | Efficacy of Elaeagnus angustifolia topical gel in the treatment of symptomatic oral lichen planus | Inadequate sample size |
| Thomas AE, Varma B, Kurup S, Jose R, Chandy ML, Kumar SP, Aravind MS, Ramadas AA | 2017 | Evaluation of efficacy of 1% curcuminoids as local application in management of oral lichen planus - interventional study | Inadequate sample size |
| Thongprasom K, Chaimusig M, Korkij W, Sererat T, Luangjarmekorn L, Rojwattanasirivej S | 2007 | A randomized-controlled trial to compare topical cyclosporin with triamcinolone acetonide for the treatment of oral lichen planus | Inadequate sample size |
| Ungphaiboon S, Nittayananta W, Vuddhakul V, Maneenuan D, Kietthubthew S, Wongpoowarak W, Phadoongsombat N | 2005 | Formulation and efficacy of triamcinolone acetonide mouthwash for treating oral lichen planus | Inadequate sample size |
| Velez I, Spielholz NI, Siegel MA, Gonzalez T | 2014 | MuGard, an oral mucoadhesive hydrogel, reduces the signs and symptoms of oral mucositis in patients with lichen planus: a double-blind, randomized, placebo-controlled pilot study | No randomization (quasi-randomized trial) |
| Veneri F, Bardellini E, Amadori F, Conti G, Majorana A | 2020 | Efficacy of ozonized water for the treatment of erosive oral lichen planus: a randomized controlled study | Inadequate sample size |
| Vohra S, Singal A, Sharma SB | 2016 | Clinical and serological efficacy of topical calcineurin inhibitors in oral lichen planus: a prospective randomized controlled trial | Inadequate sample size |
| Volz T, Caroli U, Lüdtke H, Bräutigam M, Kohler-Späth H, Röcken M, Biedermann T | 2008 | Pimecrolimus cream 1% in erosive oral lichen planus--a prospective randomized double-blind vehicle-controlled study | Inadequate sample size |
| Xiong C, Li Q, Lin M, Li X, Meng W, Wu Y, Zeng X, Zhou H, Zhou G | 2009 | The efficacy of topical intralesional BCG-PSN injection in the treatment of erosive oral lichen planus: a randomized controlled trial | Inadequate sample size |
| Zaslansky R, Schramm C, Stein C, Güthoff C, Schmidt-Westhausen AM | 2018 | Topical application of morphine for wound healing and analgesia in patients with oral lichen planus: a randomized, double-blind, placebo-controlled study | Inadequate sample size |
| Zakaria M, Said A, El-Kader AA, Mostafa B | 2020 | Evaluation of topical pomegranate extracts in management of oral lichen planus: a randomized clinical trial | Inadequate sample size |
| Zhou L, Cao T, Wang Y, Yao H, Du G, Tian Z, Tang G | 2016 | Clinical observation on the treatment of oral lichen planus with total glucosides of paeony capsule combined with corticosteroids | OLP lesions treated with systemic therapy only |
